# Metabolic control analysis enabled the improvement of the L-cysteine production process with *Escherichia coli*

**DOI:** 10.1007/s00253-023-12928-z

**Published:** 2024-01-11

**Authors:** Daniel Alejandro Caballero Cerbon, Jeremias Widmann, Dirk Weuster-Botz

**Affiliations:** Chair of Biochemical Engineering, School of Engineering and Design, Technical University of Munich, Boltzmannstr. 15, 85748 Garching, Germany

**Keywords:** L-cysteine, Fed-batch fermentation, Short-term perturbation, Metabolomics, Thermodynamic flux analysis, Metabolic control analysis

## Abstract

**Abstract:**

L-cysteine is an amino acid with relevance to the pharmaceutical, food, feed, and cosmetic industry. The environmental and societal impact of its chemical production has led to the development of more sustainable fermentative L-cysteine production processes with engineered *E*. *coli* based on glucose and thiosulfate as sulphur source. Still, most of the published processes show low yields. For the identification of further metabolic engineering targets, engineered *E*. *coli* cells were withdrawn from a fed-batch production process, followed by in vivo metabolic control analysis (MCA) based on the data of short-term perturbation experiments, metabolomics (LC–MS), and thermodynamic flux analysis (TFA). In vivo MCA indicated that the activities of the L-cysteine synthases of the cells withdrawn from the production process might be limiting, and we hypothesised that the L-cysteine precursor O-acetylserine (OAS) might be exported from the cells faster than it took to transform OAS into L-cysteine. By increasing the expression of the L-cysteine synthases, either sulfocysteine synthase or L-cysteine synthase, which transform OAS into L-cysteine, an improvement of up to 70% in specific L-cysteine productivity and up to 47% in the final L-cysteine concentration was achieved in standardised fed-batch processes thereby increasing the yield on glucose by more than 85 to 9.2% (w/w).

**Key points:**

*• Metabolic control analysis was applied to analyse L-cysteine production with E. coli*

*• OAS export was faster than its transformation to L-cysteine*

*• Overexpression of L-cysteine synthases improved L-cysteine productivity and yield*

**Supplementary Information:**

The online version contains supplementary material available at 10.1007/s00253-023-12928-z.

## Introduction

L-cysteine is a proteinogenic sulphur-containing amino acid with a global market size of approximately 5000 tons annually. It is mainly utilised as a building block in the pharmaceutical industry, as a flavour and texture additive in the food industry, as a dietary supplement in the animal feed industry, and in the cosmetic industry as an ingredient in permanent-wave products (Takagi and Ohtsu 2017). Traditionally, L-cysteine was produced by acid hydrolysis of animal hair and feathers, generating copious amounts of acidic wastewater and concerns for its human consumption due to its unreliable hygiene practices and animal origin (Hunt 1985; Berehoiu et al. 2013). For these reasons, the biotechnological production of L-cysteine, be it through whole-cell catalysis of DL-2-amino-Δ^2^-thiazoline-4-carboxylic acid with *Pseudomonas* strains (Sano and Mitsugi 1978) or through microbial fermentation with *Escherichia coli* (Dassler et al. 2000; Nakatani et al. 2012), has been increasingly investigated.

Of these processes, the fermentative biosynthesis of L-cysteine seems to be favoured using renewable and accessible feedstock and reduced sulphur sources, like thiosulfate, which minimise the cofactor (NADPH) usage in comparison to sulphate (Sekowska et al. 2000). However, the L-cysteine’s cytotoxicity at µM amounts (Sørensen and Pedersen 1991) and the complex regulatory feedback system in the L-cysteine biosynthesis constrain the L-cysteine productivity in *E. coli* (Liu et al. 2018).

Metabolic engineering approaches were applied to overcome the major metabolic roadblocks towards L-cysteine biosynthesis. These include the expression of feedback-resistant phosphoglycerate dehydrogenases and serine acetyltransferases, which are part of the L-cysteine’s precursor supply (Nakamori et al. 1998; Okuda et al. 2019). In addition, the introduction of an L-cysteine exporter was very beneficial to prevent accumulation of L-cysteine in the cytoplasm (Dassler et al. 2000; Wiriyathanawudhiwong et al. 2009). Although these approaches have substantially increased the achievable L-cysteine productivity of the engineered *E*. *coli* strains, the L-cysteine’s yield with glucose of about 6% (w/w) in the fed-batch process indicates there is still potential for optimisation (Liu et al. 2018).

Since further metabolic engineering approaches are not as straightforward due to the complex metabolic interrelationships of the L-cysteine synthesis pathway and the general carbon metabolism, a more holistic approach is necessary to identify further bottlenecks in the L-cysteine synthesis pathway of the engineered* E*. *coli* cells in the fed-batch production process.

To this end, the current research focuses on the application of the methodology of in vivo metabolic control analysis (MCA) after rapid media transition (Link et al. 2010) and short-term perturbation experiments (Weiner et al. 2016) of *E*. *coli* cells withdrawn from the production process to identify sections of the *E*. *coli* metabolism, which alteration may lead to an increase in the metabolic flux towards L-cysteine production.

MCA is a methodology that uses control coefficients to quantify the relationship between the enzyme activities and the metabolic fluxes of the reaction steps throughout a modelled metabolic pathway (Fell and Cornish-Bowden 1997). MCA takes into consideration the sensitivities of the enzymes of the metabolic model towards the cytosolic metabolite concentrations as effectors to establish how the activity of a specific enzyme, and therefore its effect in the intracellular metabolite pool, affects the metabolic flux through another enzymatic step, which must not necessarily be contiguous in the metabolic pathway (Kacser et al. 1995).

In vivo MCA after rapid media transition and short-term perturbation experiments with cells withdrawn from the fed-batch production process has already been used before for the analysis of aromatic amino acid production processes with engineered *E*. *coli* (Schoppel et al. 2021; Tröndle et al. 2018, 2020; Weiner et al. 2014). In these works, the methodologies of rapid media transition (Link et al. 2010) and short-term perturbation experiments (Weiner et al. 2016) were utilised in order to generate metabolome and fluxome databases, which were used for the calculations of MCA to identify rate-limiting steps towards the L-phenylalanine and L-tryptophan productions. Recently, Schoppel et al. showed that rational strain design based on in vivo MCA improved L-tryptophan production with *E*. *coli* (Schoppel et al. 2022).

In this study, the methodology of in vivo MCA was applied to the fermentative L-cysteine production process to identify bottlenecks in the metabolism of an L-cysteine-producing *E*. *coli* strain. To this intent, a fed-batch process was established for L-cysteine production from glucose and thiosulphate in a stirred-tank bioreactor on a 15-L scale. Afterwards, parallel short-term metabolism-perturbation experiments were carried out to obtain a database of the internal metabolome and fluxome of the cells withdrawn from the fed-batch process during L-cysteine production. Finally, this data was modelled using thermodynamic flux analysis (TFA) and MCA to characterise the effects of the enzymes of the central carbon metabolism and L-cysteine synthesis pathways on the metabolic fluxes towards L-cysteine. Identified flux-controlling enzymes were over-expressed individually in the L-cysteine-producing *E*. *coli* strain, and the improvements in process performance were studied with the standardised fed-batch process for L-cysteine production.

## Material and methods

### Strain design

The basis strain utilised for the cystine production was the *Escherichia coli* strain W3110. In order to enable the overproduction of L-cysteine, the plasmid pCys (Winterhalter and Leinfelder 1999) was transformed into wild-type cells. This plasmid contains the genes coding for tetracycline resistance with the constitutive promotor *ptetR*, the constitutive promotor *pserA1,2* regulating the expression of a feedback-insensitive phosphoglycerate dehydrogenase (Leinfelder and Heinrich 2001), a feedback-insensitive serine acetyltransferase (Bell et al. 2002) under the promotor *pcysE*, and the L-cysteine and acetylserine exporter *ydeD* with the constitutive promoter *pGAPDH* (Dassler et al. 2000).

From the results of the MCA, two new plasmids were created from the original plasmid pCys through Gibson assembly (Gibson et al. 2009). The first plasmid, named pCysK, incorporated a gene construct *cysK* for the overexpression of the enzyme L-cysteine synthetase A (CYSS) controlled by the promoter pfic1. The second plasmid, pCysM, incorporated the gene construct *cysM* for the constitutive overexpression of the enzyme L-cysteine synthase B (SLCYSS), also controlled by the promotor pfic1. The plasmid maps for these two novel plasmids are shown in Fig. S1 in the supplemental materials of this publication.

Cryo-cultures of these strains were produced by picking a single colony from an agar plate with freshly transformed cells and introducing the cell material into a 100-mL shaking flask containing 10 mL of lysogeny broth (LB) medium (Bertani 1951) with 15 mg L^−1^ tetracycline. The shaking flasks were cultivated in a shaking incubator (Infors HT, Bottmingen-Basel, Switzerland) overnight at 37 °C and 250 rpm. On the following day, 1 mL of the culture was transferred into a 250-mL shaking flask containing 23 mL of fresh LB medium with antibiotic. The cells were cultivated for 4 h at 37 °C and 250 rpm. Subsequently, 8 mL of a 60% v/v glycerine solution were added, mixed by stirring, and the resulting mixture was aliquoted into sterile 1.5-mL plastic reaction tubes (Eppendorf SE, Hamburg, Germany), which were immediately stored at − 80 °C until they were required. All the strains used in this publication are available upon reasonable request via the corresponding author.

### Production process

The preculture strategy to grow sufficient biomass for the L-cysteine production process consisted of two stages. In the first stage, 100 µL of a cryoculture from the selected strain was utilised to inoculate six 1-L shaking flasks with baffles containing 160 mL of LB medium supplemented with 10 g L^−1^ glucose and 15 mg L^−1^ tetracycline hydrochloride. The cultures were stirred in a shaking incubator for 8 h at 32 °C and 150 rpm. Afterwards, the cells were centrifuged and resuspended in 50 mL sterile phosphate buffered saline (pH 6.8, drawn into a syringe, and inoculated into the preculture reactor through a septum).

The preculture stirred-tank bioreactor (Labfors 3, Infors HT, Bottmingen-Basel, Switzerland) contained 2 L mineral medium adapted from Riesenberg et al*.* (Riesenberg et al. 1991), which consisted of 5 g L^−1^ (NH_4_)_2_SO_4_, 5 g L^−1^ KH_2_PO_4_, 1 g L^−1^ trisodium citrate dihydrate, 0.5 g L^−1^ NaCl, 75 mg L^−1^ FeSO_4_·7H_2_O, and 10 mL L^−1^ of a trace element solution containing 3.75 g L^−1^ H_3_BO_3_, 1.55 g L^−1^ CoCl_2_·6H_2_O, 0.55 g L^−1^ CuSO_4_·5H_2_O, 3.55 g L^−1^ MnCl_2_·4H_2_O, 0.65 g L^−1^ ZnSO_4_·7H_2_O, and 0.33 g L^−1^ Na_2_MoO_4_·H2O. The reactor and the mineral medium were autoclaved at 121 °C for 20 min. 1.2 g L^−1^ MgSO4·7H2O, 1 g L^−1^ threonine, 0.9 g L^−1^ isoleucine, 0.6 g L^−1^ methionine, 0.23 g L^−1^ CaCl_2_·2H_2_O, 90 mg L^−1^ pyridoxine hydrochloride, 18 mg L^−1^ thiamine hydrochloride, and 15 mg L^−1^ tetracycline hydrochloride were sterile-filtered and added to the stirred-tank bioreactor after autoclaving. A 600 g L^−1^ glucose solution was autoclaved separately and added to the reactor to reach a concentration of 40 g L^−1^. The preculture reactor was operated for 17–18 h at 32 °C, aeration of 4 NL sterile air min^−1^ (2 vvm), and varying stirrer speeds to keep the dissolved oxygen content above 30% air saturation. In the stirred-tank bioreactor, pH 7.0 was controlled online by titration with 25% (w/w) NH_4_OH or 13% (w/w) H_3_PO_4_. Antifoam agent (Antifoam 204, Merck, Darmstadt, Germany) was supplied as required through an automated foam protection system. Once the reactor reached a biomass concentration over 18 g L^−1^ dry cell mass, the stirred-tank bioreactor content was sterilely pumped out of the reactor into a sterile flask and used as inoculum for the L-cysteine production process.

For the L-cysteine production process, 9 L of the same mineral medium described above was autoclaved in place in the stirred-tank bioreactor (Techfors 42 L, Infors HT, Bottmingen-Basel, Switzerland). The medium was supplemented as described above, except for the initial glucose concentration being 10 g L^−1^ in the production reactor. Subsequently, the inoculum was pumped into the reactor to reach a biomass concentration of 1.8 g L^−1^ dry cell mass. To reach an initial reactor volume of 10 L, the missing volume was filled up with reverse osmosis water.

The L-cysteine production process was started at 32 °C, 1.7 bar, aeration of 20 NL min^−1^ with pressurised air, and stirring at 300 rpm with 2 Rushton turbines. The stirrer speed was adapted by a PI controller to keep the dissolved oxygen concentration at 40% air saturation. pH was maintained at pH 7.0 by automatic titration of 25% (w/w) NH_4_OH, or 13% (w/w) H_3_PO_4_ Antifoam agent (Antifoam 204, Merck, Darmstadt, Germany) was supplied as required through an automated foam protection system. Two and a half hours after inoculation, a sterile 30% (w/w) ammonium thiosulfate solution started being fed to the reactor at a rate of 10 g h^−1^. The thiosulfate feeding rate profile can be found in Fig. S2 of the supplemental materials together with the glucose feeding profile, which started once the initially provided glucose was fully consumed in the batch process, as evidenced by a surge in the dissolved oxygen concentration, and had a glucose feed concentration of 670 g L^−1^. These feeding profiles follow a heuristic approach, which has been proven to prevent substrate accumulation in the stirred-tank bioreactor. Samples were taken during the daytime every 1.5 h through a sampling valve at the bottom of the stirred-tank bioreactor and frozen at − 20 °C until subsequent analysis.

### Short-term experiments

The L-cysteine production process presented above served as a reference process for a parallel steady-state metabolism perturbation experiment. During the productive phase of the 10 L-scale process, around 22 h after inoculation, a 4-L sample was withdrawn from the production reactor through a valve at the bottom of the reactor. This sample was handled following a rapid media transition protocol (Link et al. 2010). The 4-L sample was centrifuged immediately after sampling at 32 °C and 3260 g for 7.5 min (Rotixa 50 RS, Andreas Hettich GMBH & Co., Tuttlingen, Germany) in custom-made centrifuge vessels with a 3D-printed mesh (details are shown in Fig. S3 in the supporting information of this publication) that enabled the rapid separation of the biomass and cystine pellets once the supernatant was discarded. The biomass portion of the pellet was resuspended in fresh tempered (32 °C) mineral medium without glucose and supplemented with 9 g L^−1^ (NH_4_)_2_S_2_O_3_, which would serve as inoculum for the parallel four 500 mL stirred-tank bioreactors (fourfold DASGIP® Bioblock-System for Microbiology, Eppendorf SE, Hamburg, Germany) that was used for the short term experiments. The 400 ml cell suspension was distributed evenly to the four reactors, which already contained 400 ml tempered mineral medium without a carbon source. The parallel fed-batch cultivations were performed at 32 °C, with aeration of 4 NL min^−1^ of a mixture of pressurised air and pure oxygen, resulting in an inlet oxygen share of 25% (v/v), stirring at 1200 rpm with two 6-bladed Rushton impellers. The pH was maintained at pH 7.0 by adding 2 M NaOH or 21% (w/w) H_3_PO_4_.

In order to generate internal metabolome and extracellular fluxome databases for the MCA, it was necessary to instigate deviations in the metabolism from the reference L-cysteine production process and generate metabolic steady states to ensure that the metabolic pathways have reached an equilibrium (Weiner et al. 2016). Additionally, for the calculation of control coefficients, the enzyme levels must remain unchanged, a state sought after through a 23-min cultivation since the enzyme concentrations are not expected to change considerably in such a short time interval (Link et al. 2010). During this cultivation, each reactor was fed with a different carbon source, i.e. glucose (35 g L^−1^), pyruvate (52 g L^−1^), a mixture of glucose (39 g L^−1^) and pyruvate (32 g L^−1^), and a mixture of glucose (28 g L^−1^) and succinate (18 g L^−1^), at three increasing feeding rate steps of 30, 60, and 90 mL h^−1^. The feed concentrations were chosen in order to balance the maximal substrate uptake rate, which was determined preliminarily (data not shown), at the highest feed rate. Each of the feeding steps lasted for 7 min, which has been shown to be sufficient time for the *E*. *coli* metabolism to reach a pseudo-steady state under these conditions (Link et al. 2012; Schoppel et al. 2022; Tröndle et al. 2020; Weiner et al. 2016). During the short-term experiments, samples were taken from each reactor at the beginning and directly before the end of each feed rate step using a custom-made sampling system (Hiller et al. 2007), which allows for simultaneous sampling and instantaneous metabolic inactivation (quenching of the metabolism) by dispersing the sample into a 100% methanol / 30 mM aqueous triethanolamine solution (60:40) at − 54 °C. The sample tubes with the quenched samples were stored immediately after sampling at − 20 °C.

Once the short-term cultivations ended, 1 mL of every inactivated sample was added to 2 mL of tempered 30 mM triethanolamine at 95 °C for 5 min to extract the internal metabolites from the inactive cells. After cooling on ice for 15 min, the extracted samples were centrifuged at 3260 g for 10 min at 4 °C (Rotixa 50 RS, Andreas Hettich GMBH & Co, Tuttlingen, Germany). This process was carried out in quadruplets for each sample, where two replicates were supplemented with 335 µl of a U-^13^C-labelled cell extract prepared as described by Weiner et al. (2015) as internal standard (once undiluted and once diluted 1:10). The supernatant was stored at − 80 °C until its analysis with LC–MS.

### Analytical determinations

Online process data was collected using the software IRIS V5.3 (Infors HT, Bottmingen-Basel, Switzerland) for the reference fed-batch process and Dasgip Control (Eppendorf SE, Hamburg, Germany) for the short-term parallel experiments.

The biomass concentration was determined gravimetrically in triplicates. For this, 1 mL of a process sample was pipetted into reaction tubes previously dried for 72 h and weighed. The samples were then centrifuged (Centrifuge 5424 R, Eppendorf SE, Hamburg, Germany), and the supernatant was separated for use in the high-performance liquid chromatography (HPLC) analyses described below. A total of 1 mL of a solution containing 12% (v/v) H_3_PO_4_ and 1.5% H_2_SO_4_ (v/v) was added to the reaction tube containing the pellet to dissolve the precipitated cystine by vortexing the sample for 10 min. The reaction tubes were centrifuged again, and the supernatant was again captured for HPLC determinations. The reaction tubes with the cell pellet were dried in a drying oven (UN 260, Memmert GmbH, Schwabach, Germany) at 80 °C for at least 48 h and weighed. The biomass concentration results from the difference in dry weight between the empty and the full reaction tube over the sample volume.

The concentrations of glucose, phosphate, malate, pyruvate, succinate, formate, acetate, and ethanol were determined via HPLC (Agilent Series 1100, Agilent Technologies, Santa Clara, USA). A total of 20 µL of the sterile-filtered supernatant from the sample were injected into an ion exchange column (Aminex HPLX-87H, Bio-Rad, Munich, Germany) at 65 °C with an isocratic carrier flow of 0.7 mL min^−1^ of 5 mM H_2_SO_4_. Each sample run took 32 min. The samples were measured using a refractive index detector (Agilent Series.1100, Agilent Technologies, Santa Clara, USA) at 950 nm.

L-cysteine, L-cystine, N-acetylserine, and thiosulfate concentrations were determined through HPLC (Dionex Ultimate 3000, Thermo Fisher Scientific, Waltham, USA). Thiosulfate, acetylserine, and L-cysteine were measured from the supernatant after centrifugation of the reactor sample. Cystine was measured from the supernatant of the resuspended cystine pellet in an acidic mixture as described above. A total of 2 µL of the sample were injected into a reverse phase separation column (Gemini 5 µm C18 110 Å 250X4.6 mm, Phenomenex, Torrance, USA) at 20 °C using a carrier stream of 0.5 mL min^−1^ of a solution containing 3.4% (v/v) H_3_PO_4_ and 0.04% (v/v) H_2_SO_4_. The samples were measured with a UV–Vis detector (DAD-3000, Thermo Fisher Scientific, Waltham, USA) at 200 nm and 210 nm. After the measurement, which took 20 min for each sample, the column was regenerated with an elution flow of 1 ml min^−1^ pure acetonitrile for 5 min and equilibrated for 10 min at 0.5 mL min^−1^ with the acidic eluent.

The LC–MS (Autosampler: Thermo Pal; Pump: Accela; Oven: MistraSwitch; Detector: TSQ Vantage, Thermo Fisher Scientific, Waltham, USA) analysis was carried out following the methodology described by Buescher et al. (2010). A total of 20 µL of the extracted metabolite sample was injected into a C18 UHPLC separation column (Acquity HSS T3, Waters Corporation, Milford, USA) at 40 °C. A 36-min gradient of solvent A (10 mM tributylamine, 15 mM acetic acid, 5% (v/v) methanol) and solvent B (100% isopropanol) was utilised for the metabolite elution. The analytes were ionised with an electro-sprayer at a voltage of 2.8 kV, a capillary temperature of 380 °C, and a vaporiser temperature of 400 °C. The metabolites were detected on the first quadrupole of a triple quadrupole detector on its negative polarity operation (TSQ Vantage, Thermo Fisher Scientific, Waltham, USA).

### Metabolism modelling

The cysteine production process’ genome-scale thermodynamic-based flux analysis (TFA) was performed using the Python package pyTFA (Salvy et al. 2018). The *E*. *coli* iJO1366 genome model included in the Python package was utilised as a base. The missing reactions catalysed by the enzymes sulfo-cysteine synthase, sulfo-cysteine lyase, sulphite reductase, and thiosulfate sulfurtransferase were added to the model. The identifiers, molecular weight, pKa, structure cues, and ΔG_f_ of the metabolite S-sulfo-l-cysteine were also added. The Δ*G*_f_ was calculated using the group contribution methodology developed by Mavrovouniotis (1990).

Flux distributions were calculated for the reference L-cysteine production process and every equilibrium stage achieved during the short-term experiments. The TFA workflow included a loopless flux variance analysis, which eliminated biologically unfeasible flux loops and calculated flux distributions to 99.9% of the objective’s function maximal value. The measured extracellular rates of substrate uptake, (by)product formation, and respiration, as well as the internal metabolite concentrations, were utilised as constraints to reduce the solution space. Maximising the growth rate was used as the optimisation’s objective function.

The TFA analysis provided an optimal thermodynamically feasible flux distribution as well as ranges for every Gibbs reaction energy, reaction rate, and metabolite concentration in the model. For the MCA, these results were used in a reduced metabolic model comprising 37 reactions and 40 metabolites of the glycolysis, pentose phosphate, citrate cycle, and L-cysteine synthesis pathways. This reduced model is illustrated in Fig. S4 of the supplementary information.

The MCA was performed with Matlab R2021a (Mathworks, Natick, USA). The elasticity coefficients were calculated using either the thermokinetic affinity model of Nielsen (1997) for reactions near thermodynamic equilibrium or the lin-log approach of Visser and Heijnen (2002) for reactions far from equilibrium. The equations presented by Wang et al. (2004) were used for control coefficient calculations. Following the protocol of this last group, a Monte Carlo sampling algorithm (10,000 cycles) was utilised to account for uncertainties in the measurement and calculation of the metabolite concentrations, extracellular fluxes, and Gibbs reaction energies.

## Results

### Reference production process

**Fig. 1:**
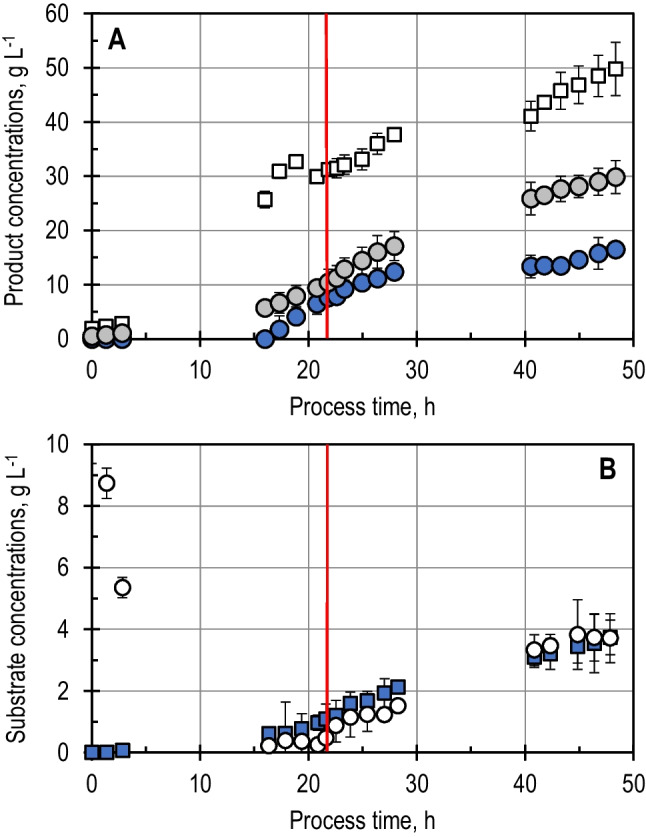
L-cysteine fed-batch production process with *E. coli* W3110 pCys on a 15-L scale (reference process). **A** Biomass (*squares*), L-cysteine (*blue circles*), and N-Acetylserine (*grey circles*) concentration profiles. The red line indicates the process time when the cells are sampled to perform short-term perturbation experiments. **B** Substrate concentration profiles. Glucose (*circles*) was supplied both initially and via feeding. Thiosulfate (*blue squares*) was fed beginning 2 h after inoculation. The exact feeding rate profiles are provided in the supplementary information. The stirred-tank reactor was operated with 2 Rushton turbines, 32 °C, 1.7 bar, aeration with 20 NL min^−1^ sterile air, DO > 40% air saturation, stirred speed 300–1000 rpm, and pH 7.0. The fed-batch process was reproduced 5 times

The L-cysteine production process with *E*. *coli* strain W3110 pCys was characterised on a 15-L scale in a stirred-tank bioreactor. The fed-batch process was carried out in quadruplicate to account for process reproducibility during its characterisation and an additional time to generate the inoculum for the short-term experiments. Figure 1A presents the volume and the biomass, L-cysteine and, as the major by-product of the fermentation, N-acetylserine (NAS) concentrations of the fed-batch L-cysteine production process used as a reference for the short-term experiments. Figure 1B presents the substrate (glucose and thiosulfate) concentrations in the reactor medium.

The stirred-tank bioreactor was consistently inoculated with 1.8 g L^−1^ biomass (dry cell mass). The *E*. *coli* cells immediately started growing exponentially until 20 h after inoculation. Afterwards, a further increase in the biomass concentration was observed, which lasted until the end of the fed-batch cultivation. The end of the exponential growth phase seems to align itself with the initiation of the L-cysteine production phase, which started 16 h after inoculation once the thiosulfate concentration in the medium increased. The NAS concentration began to increase during the exponential growth phase and reached a concentration of 29.9 g L^−1^ by the end of the process. The maximal achieved L-cysteine concentration was 16.2 g L^−1^, reached 48 h after inoculation. The maximal biomass-specific productivity of this process was 16.5 mg g_X_^−1^ h^−1^, observed 21 h after inoculation.

The batch phase of these processes lasted 4–5 h up to the complete consumption of the initially added glucose, whose concentration declined rapidly once the cells were added. The thiosulfate and glucose feeding rates were selected to ensure minimal accumulation of the substrates, resulting in a maximal concentration of 3.8 g L^−1^ of each component.

Since the objective of the flux analyses was to investigate possible rate-limiting steps and allosteric effects on the L-cysteine synthesis pathway, the sampling of 4 L of the reactor content was performed during the phase of maximal biomass-specific L-cysteine productivity of the reference process 21 h after inoculation.

### Short-term experiments

After the rapid media transition, the four parallel stirred-tank bioreactors were inoculated to achieve an initial biomass concentration of 33 g L^−1^ dry cell mass. The high biomass concentration is necessary to better observe changes in the metabolite concentrations within the short-term analyses of 23 min in total. The four parallel stirred-tank bioreactors were fed with (1) glucose, (2) pyruvate, (3) a mixture of glucose and pyruvate, and (4) a mixture of glucose and succinate at three increasing feeding rate steps to achieve 12 metabolic steady states.

**Fig. 2:**
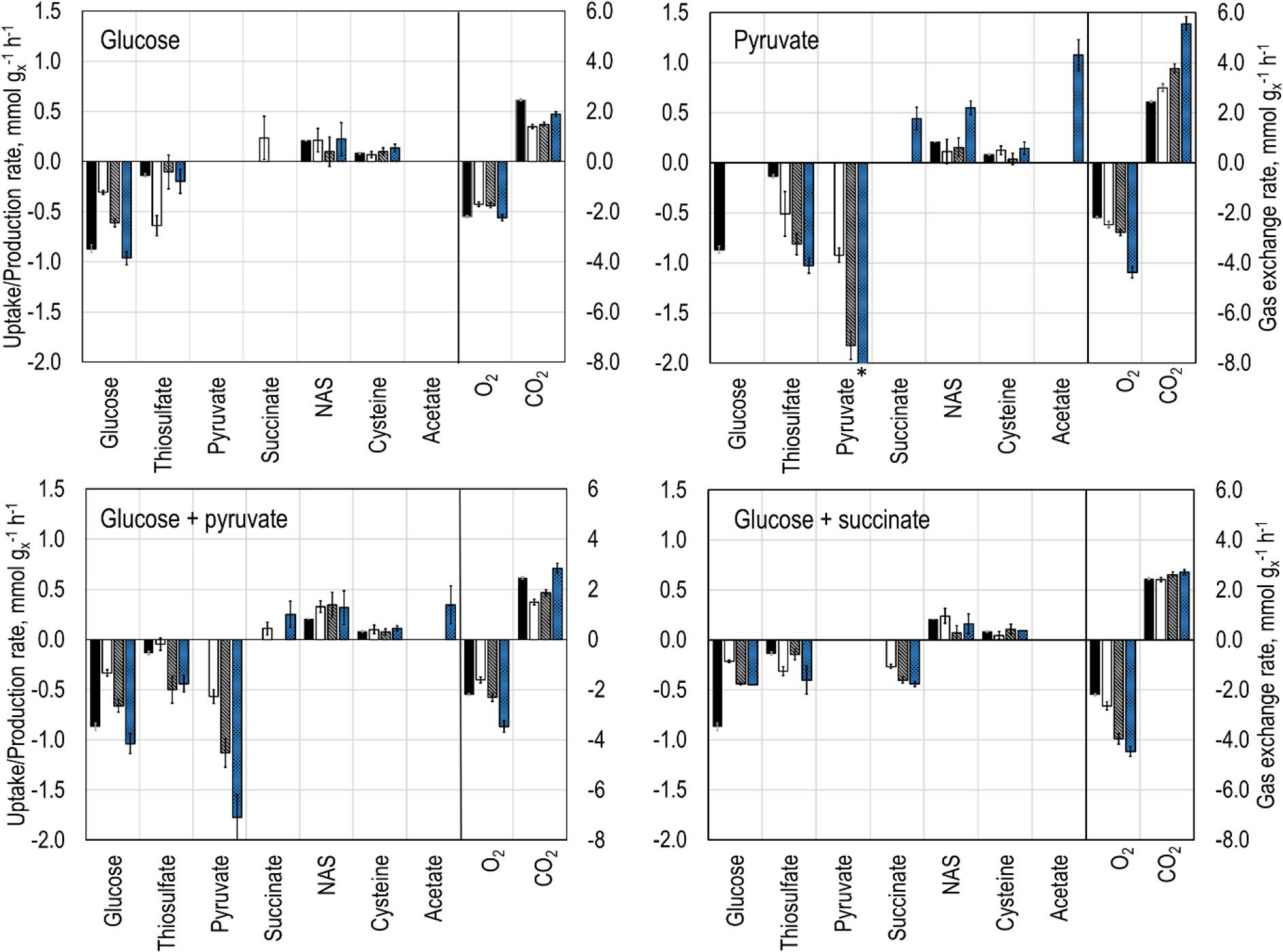
Extracellular transport rates measured during the parallel short-term experiments performed with *E. coli* W3110 pCys. In this experiment set, four different reactors were inoculated with a sample harvested from the L-cysteine production process on a 15-L scale. Subsequently, different substrate sources were fed to each stirred-tank bioreactor. Stocks of glucose (*top left*), pyruvate (*top right*), a mixture of glucose and pyruvate (*bottom left*), and a mixture of glucose and succinate (*bottom right*) were fed to the reactors in three increasing steps of 30 (white bars), 60 (grey bars), and 90 ml h^−1^ (blue bars) with a step-time of 7 min. The data of the L-cysteine production process, where the sample was taken from, is also shown (black bars). The parallel stirred-tank reactors were operated with 2 Rushton turbines, 32 °C, 1 bar, aeration with 4 NL min^−1^ sterile air, DO > 40% air saturation, stirred speed 1200 rpm, and pH 7.0. Error bars indicate the measurement inaccuracy of three technical replicates (* − 2.87 mmol g_x_^−1^ h^−^.^1^)

The difference in the concentrations of metabolites in the medium between the samples taken at the beginning and at the end of each feeding rate step was used to calculate the extracellular transport rates. The biomass-specific substrate uptake rates, product and by-product formation rates, and respiration rates are presented in Fig. 2.

It was possible to observe increasing uptake rates for all C-sources supplied to the four parallel-operated stirred-tank bioreactors, corresponding to the three feeding rate steps. Throughout the experiments, no substrate accumulation was measured in the medium. Although the L-cysteine and NAS production, coupled with thiosulfate uptake, was observed in all metabolic equilibrium stages, no clear trend could be identified for these rates. Acetate and succinate production was observed in the reactors supplied with pyruvate at the higher feeding rates in the later equilibrium stages.

### Metabolic analyses

The extracellular rates and the internal metabolite concentrations of the reference process at the time of the sample withdrawal and of the metabolic equilibrium stages of the short-term perturbation experiments (which can be found in Table S1 of the supplementary information) were introduced in the pyTFA routine to generate 13 different metabolic flux distributions, which are presented in Table S2 of the supplementary information for reactions of selected metabolic pathways. Additionally, to the flux distributions, the TFA also provided the Gibbs free energy of reaction for all the reactions in the model according to the supplied or calculated metabolite concentrations. This provided information about how close the given reactions were to equilibrium. This information is essential for the MCA because the method of elasticity calculation for each reaction varies depending on its distance from equilibrium.

**Fig. 3:**
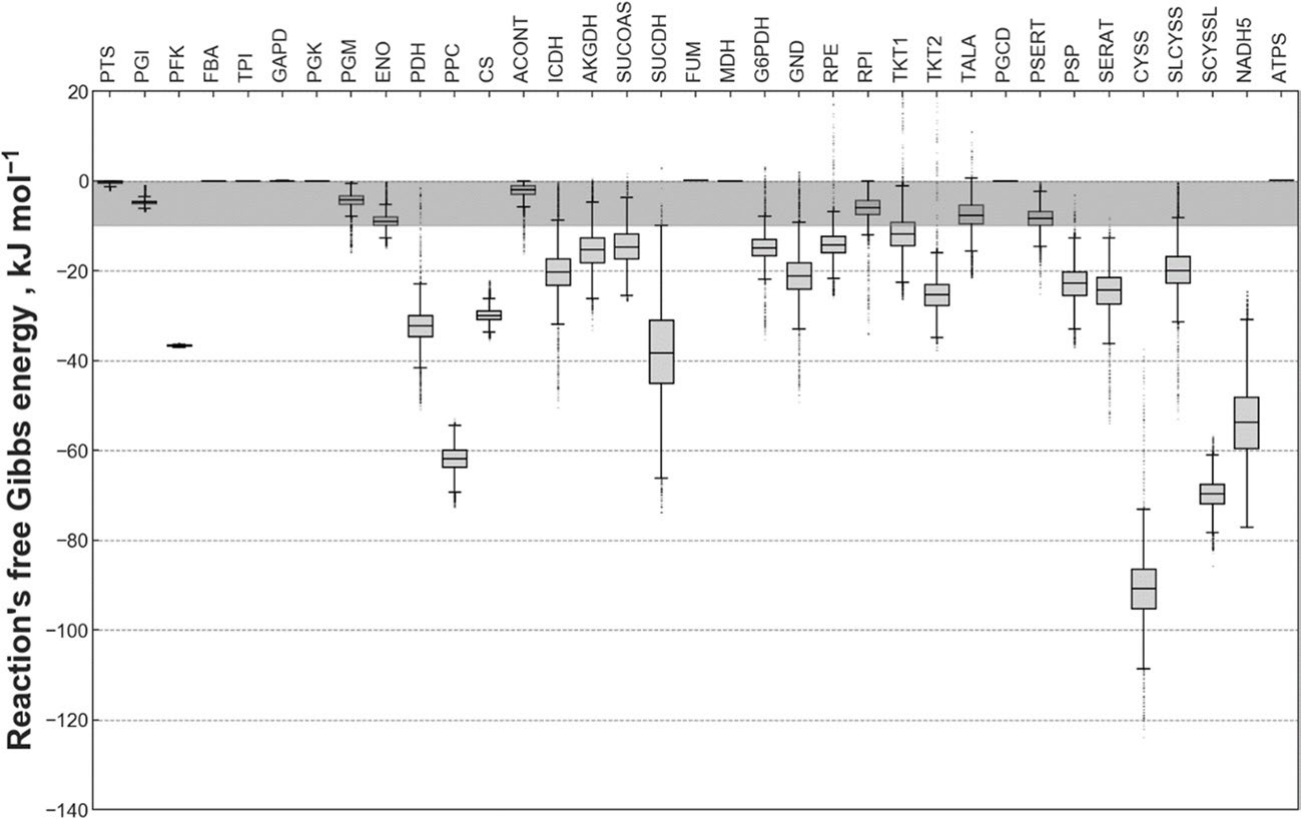
Estimated free Gibbs energies of the reactions used for the metabolic control analysis (MCA). The presented distributions originate from a 10,000-iteration Monte-Carlo sampling algorithm applied to the thermodynamic flux balance of the genome-wide flux distribution from the reference process. Reactions whose two middle quartiles lie above − 10 kJ mol^−1^, indicated by a grey area in the figure, were considered to be in equilibrium for the MCA. A list of the abbreviations for the reactions in this figure can be found at the end of this publication

An example of the resulting Gibbs free energy ranges is observable in Fig. 3 for selected TFA reactions performed on the reference process’ fluxome and metabolome data. The observed distributions are the results of the Monte Carlo sampling algorithm of the metabolic fluxes and metabolite concentrations within the minimal and maximal boundaries obtained from the TFA for the reference process. A reaction was determined to be in equilibrium if its two middle quartiles lay between 0 and − 10 kJ mol^−1^. For the MCA, the following reactions were considered to be in equilibrium: glucose 6-phosphate Isomerase (PGI), fructose bisphosphatase A (FBA), triose-phosphate isomerase (TPI), glyceraldehyde 3-phosphate dehydrogenase (GAPD), phosphoglycerate mutase (PGM), enolase (ENO), aconitase (ACONT), fumarase (FUM), malate dehydrogenase (MDH), ribose-5-phosphate isomerase (RPI), transaldolase (TALA), phosphoglycerate dehydrogenase (PGCD), phosphoserine transaminase (PSERT), and ATP synthase (ATPS). The glucose transport via the pyruvate transfer system (PTS) is considered not to be in equilibrium due to the irreversible nature of the phosphorylation reaction chain. The following reactions were considered not to be in equilibrium for the elasticity calculations since their Gibbs reaction energy was below − 10 kJ mol^−1^: phosphofructokinase (PFK), phosphoserine phosphatase (PSP), pyruvate dehydrogenase (PDH), phosphoenolpyruvate carboxylase (PPC), citrate synthase (CS), isocitrate dehydrogenase (ICDHyr), α-ketoglutarate dehydrogenase (AKGDH), succinyl-CoA synthetase (SUCCOAS), succinate dehydrogenase (SUCDH), glucose-6-phosphate dehydrogenase (G6PDH), phosphogluconate dehydrogenase (GND), ribulose 5-phosphate 3-epimerase (RPE), both transketolases (TKT1 and TKT2), serine acetyltransferase (SERAT), L-cysteine synthase (CYSS), sulfo-l-cysteine synthase (SLCYSS), sulfo-l-cysteine lyase (SCYSSL), and NADH dehydrogenase (NADH5).

### Metabolic control analysis

**Fig. 4:**
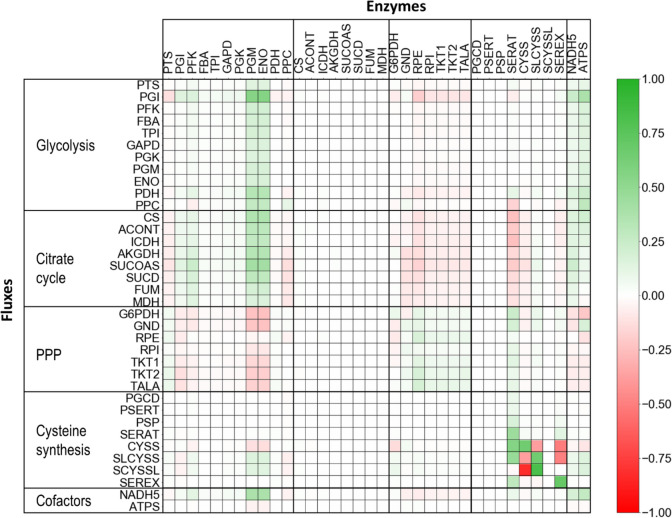
Metabolic control map for the L-cysteine production with *E*. *coli* pCys cells withdrawn from the fed-batch production process at a process time of 22 h. The values in the cells indicate the degree of control that the enzymatic activity of a specific metabolic step (*columns*) assert over the metabolic flux through another reaction (*rows*). A positive value indicates that increasing the enzymatic activity of the step in the cell’s column would lead to a higher flux through the reaction in the cell’s row and vice versa

The results of the in vivo MCA provide insights into the metabolic control that certain enzymatic steps exert over the metabolic fluxes through other reactions and, most relevant for this research, over the L-cysteine synthesis pathway of *E*. *coli* cells withdrawn from the reference fed-batch production process at a process time of 22 h at high cell-specific L-cysteine productivity. The calculated flux control coefficients (FCCs) for the reduced metabolic model are summarised in Fig. 4. The control coefficients are dimensionless since the fluxes and metabolite concentrations used for their calculation have been normalised through division over their respective values of the reference process (Fell 1992). The value of the control coefficients may be understood as the effect of a 1% increase in the activity of an enzyme on a specific metabolic flux in the pathway.

The strong effects of the enzymatic activities of PGM and ENO (Fig. 4) on the metabolic fluxes throughout the glycolysis, citrate cycle, pentose phosphate pathway, and, to some lower extent, the L-cysteine synthesis pathway have been reported previously for other *E*. *coli* strains (Andreozzi et al. 2016; Schoppel et al. 2022) and suggest that these reactions are rate-limiting and play an essential role in the carbon distribution of the central carbon pathways.

The focus of this research is the metabolic fluxes through the L-cysteine synthesis pathway, which according to the results of the in vivo MCA are predominantly controlled by their respective enzymatic activities (Fig. 4). *E*. *coli* cells are capable of producing L-cysteine from O-acetylserine and sulphate through the L-cysteine synthase (CYSS) or with thiosulfate through the sulfocysteine synthase (SLCYSS). In the latter case, the OAS and thiosulfate are transformed into sulfocysteine and a second enzyme, sulfocysteine lyase (SCYSSL), cleaves the thiol bond generating L-cysteine and a sulphite molecule that can be incorporated into a second L-cysteine molecule via CYSS (Sekowska et al. 2000). The L-cysteine synthases, CYSS and SLCYSS, show simultaneously the highest positive control coefficient on themselves and the most negative control coefficient on each other. This indicates a competitive relationship between these two enzymes while at the same time suggesting they are rate-limiting for L-cysteine production.

The fact that the upstream reaction SERAT holds a positive control despite already being over-expressed without feedback inhibition, together with the competitive relationship described above for the L-cysteine synthases, which have OAS as a common substrate, seem to indicate that the intracellular accessibility of OAS is limiting the L-cysteine production The leading cause of reduced intracellular availability of OAS may be its export through the same exporter used for L-cysteine secretion (*ydeD*/SEREX, Franke et al. 2003). Further cementing this hypothesis is the negative control coefficient of SEREX on CYSS and SLCYSS, indicating that the exporter removes the OAS from the cell before the L-cysteine synthases have the chance to transform it into L-cysteine.

A slight degree of control from G6PDH over the L-cysteine synthases and the sulfocysteine sulphite lyase (SCYSSL) is explained by the role of G6PDH in the NADPH generation. SCYSSL utilises the cofactor to transform sulfocysteine in L-cysteine and sulphite. Since NADPH is only required in the thiosulfate branch of L-cysteine synthesis, and due to the competitive relationship between both synthases, G6PDH has a positive control on SLCYSS and SCYSSL and a negative control coefficient on CYSS (Fig. 4).

### Strain optimisation based on the MCA results

From the results of the in vivo MCA, strain optimisation strategies were formulated to circumvent an intracellular OAS limitation that reduces the L-cysteine yield. Since the genes for the overexpression of a feedback-insensitive SERAT are already in the plasmid, and the exporter SEREX is necessary for the L-cysteine export, the decision was made to overexpress the L-cysteine synthases individually.

**Fig. 5:**
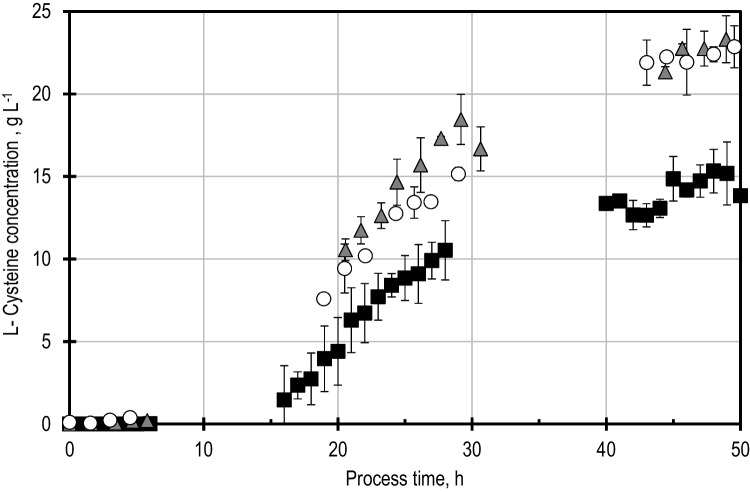
Comparison of the L-cysteine concentration profiles of the standardised fed-batch production processes with *E. coli* W3110 carrying different plasmids. The fed-batch process with the cells carrying plasmid pCys (*squares*) is used as reference, and its profile was generated by plotting a 1.5-h moving-window average of 5 production processes with this strain because sampling was not exact at the identical process times. The processes with cells carrying the pCys plasmid with an additional gene for the overexpression of L-cysteine synthase (*triangles*) or sulfocysteine synthase (*circles*) are presented as representative single processes for ease of view.The error bars for these two profiles represent the standard deviation of the analytical replicates. The biological replicates with the strains overexpressing L-cysteine synthase and sulfocysteinsynthase can be found in Figs. S5 and S6 in the supplementary information. The feeding rate profiles are provided in the supplementary information. The stirred-tank reactor with an initial volume of 10 L was operated with 2 Rushton turbines, 32 °C, 1.7 bar, aeration with 20 NL min^−1^ sterile air, DO > 40% air saturation, stirred speed 300–1000 rpm, and pH 7.0

The pCys plasmids also carrying either the gene *cysK* for CYSS overexpression or the gene *cysM* for SLCYSS overexpression were transformed into wild-type *E*. *coli* W3110 cells. Standardised L-cysteine fed-batch production processes were carried out on a 15-L scale with these new strains. In Fig. 5, the resulting L-cysteine concentration profiles with these strains are compared to the original L-cysteine production process with *E*. *coli* W3110 pCys. The L-cysteine production processes with the L-cysteine synthase-overexpressing mutants were carried out in triplicate to investigate the reproducibility of the results and for statistical evaluation. The maximal specific L-cysteine productivity increased by 8.3% for the overexpressing *E*. *coli* mutant SLCYSS (17.9 mg g_x_^−1^ h^−1^) and by 69.8% for the *E*. *coli* mutant overexpressing CYSS (28.1 mg g_x_^−1^ h^−1^). The yield on glucose increased as well from 4.96% of the supplied carbon from glucose ending up as L-cysteine in the reference fed-batch process to 7.99% with the SLCYSS overexpressing *E*. *coli* mutant and 9.21% with the CYSS overexpressing *E*. *coli* mutant, which represents an increase above 85% compared to the reference process).

**Fig. 6:**
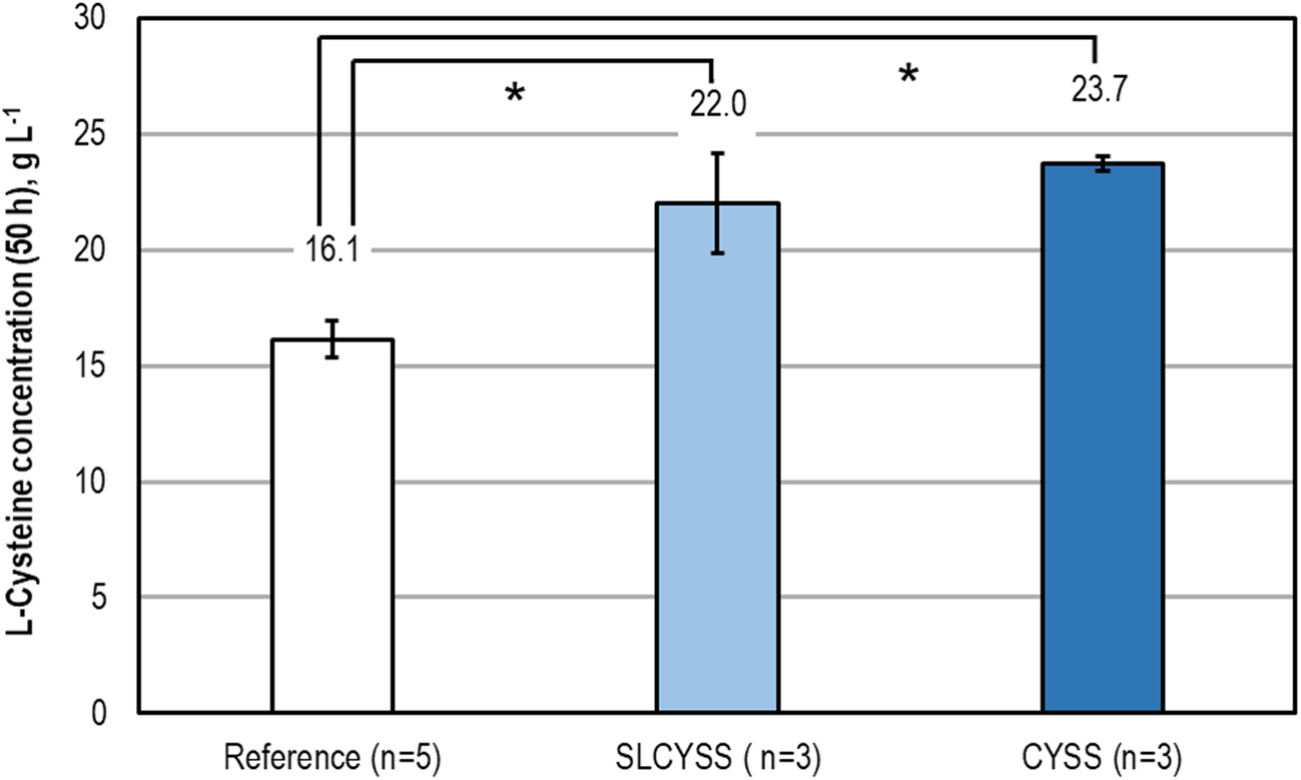
Comparison of the maximal L-cysteine concentration after 50 h of the fed-batch production processes with *E*. *coli* W3110 carrying different plasmids. The error bars indicate the standard deviation of the 5 replicates for the reference process and 3 replicates for the processes with the plasmids that overexpressed either sulfocysteine synthase (SLCYSS) or L-cysteine synthase (CYSS). *p = 0.05 in a single-tailed Student *t* test

Figure 6 presents the maximal achieved L-cysteine concentration for the reference fed-batch process with pCys and both synthase-overexpressing mutants. A single-tailed Student *t* test was performed between the reference process and each fed-batch process with synthase overexpressing mutants. The L-cysteine concentration 50 h after inoculation of the synthase overexpressing mutants is significantly (*p* < 0.05) higher than that of the reference process. The SLCYSS overexpressing mutant reached a maximal L-cysteine concentration of 20.4 ± 2.2 g L^−1^, and the CYSS overexpressing mutant a maximal concentration of 23.7 ± 0.3 g L^−1^.

## Discussion

In vivo MCA of *E*. *coli* cells withdrawn from the fed-batch production process at high specific productivity delivered a snapshot of the complex regulatory network of the central carbon metabolism and its interconnections with the L-cysteine synthesis pathway. The application of the obtained insights into strain optimisation resulted in a specific productivity increase of up to 69.8% and an increase in the maximal L-cysteine concentration of up to 47.2%. It also presented a comprehensible data-driven explanation for the N-acetylserine accumulation observed in the reference fed-batch process since OAS reacts to N-acetylserine under process conditions outside of the *E. coli* cells. The exporter *ydeD* was found to drain the intracellular OAS reserves before the L-cysteine synthases were able to transform it into L-cysteine, negatively impacting the L-cysteine production.

The export of OAS by the gene product of *ydeD* was first described in the publication that characterised this gene and its gene product (Dassler et al. 2000). Franke et al. (2003) proposed an alternative exporter, *yfiK,* which also transports L-cysteine and OAS out of the cell with a lower affinity than *ydeD*. As consequence of this lower affinity, the export of L-cysteine and OAS with the gene product of *yfiK* was only reported when the OAS synthesis pathway was upregulated. Another alternative may be the overexpression of the exporter *bcr* (Yamada et al. 2006), which was shown to export L-cysteine more efficiently than *ydeD* and has high selectivity for leucine, valine, proline, serine, arginine, glutamic acid, and methionine, but its selectivity for OAS is not described. Protein engineering approaches to increase the selectivity of *ydeD*, *yfiK*, or *bcr* towards L-cysteine may be the key to preventing the depletion of intracellular OAS.

As mentioned before, one of the principal requirements for the MCA is that the enzyme concentrations remain unchanged during the short-term experiments where the metabolome and fluxome data is gathered. This is of particular importance for the targets of the MCA that were modified during strain optmization. Mino et al. (2000) determined that L-cysteine synthase from *E*. *coli* JM 70 retained 100% of its enzymatic activity for at least 12 h when incubated in a phosphate buffer at pH 7.5 and 30 °C. Vahidi et al. (2016) immobilised *E*. *coli* MG1655 CYSS and SLCYSS for the transformation of OAS and pyrazole to ß-pyrazol-1-yl-L-alanine. After ten rounds of 20 min of continous transformation at pH 7 and 35 °C, the enzymes retained 57 and 54% of their initial activity, respectively.

The reason for the disparity of improvement in the L-cysteine productivity and final L-cysteine concentrations between the strain overexpressing CYSS and the strain overexpressing SLCYSS is most likely the sulphur utilisation. While the overexpression of SLCYSS intensifies a pathway already being utilised for L-cysteine synthesis from thiosulfate and only utilises one of the two sulphur atoms of thiosulfate, the overexpression of CYSS enhances a parallel synthesis pathway that can take advantage of the second sulphur atom of thiosulfate after is cleavage from sulfocysteine by SCYSSL. This is supported when comparing the L-cysteine’s sulphur molar yields of the production processes with these strains; the overexpression of SLCYSS increased the sulphur molar yield of L-cysteine from up to 22.7% in the reference process with pCys to up to 55.0% with the strain carrying the plasmid pCysM. This means that with the overexpression of SLCYSS slightly more than half of the sulphur atoms provided to the process are incorporated into cysteine. In contrast, the overexpression of CYSS increases the sulphur molar yield to up to 74.7%. This effect was not quantified in the MCA because the model does not take the availability of sulphur ions into account, since it was not possible to quantify the intracellular sulfphur ion concentrations with the available analytical methods.

The use of in vivo MCA may lead to more bioprocess-relevant and more targeted strain optimisation approaches, as was the case in the present publication. However, they require extensive knowledge of the enzyme regulation and allosteric effects caused by the internal metabolites to accurately model the impact of deliberate changes in the enzyme levels on a genome-wide scale. The integration of kinetic information is required to understand the mechanism underlying the change in metabolic fluxes that can be calculated by constraint-based models like MCA and remains a topic of great scientific interest (Link et al. 2014). As an example, Bi et al. (2023) utilised a small kinetic model of glycolysis to accurately predict oscillations in the internal pyruvate concentrations in starved *E*. *coli* cells which were submitted to stepwise increases in the feeding rates of glycolytic intermediates. The integration in the genome-scale metabolic modelling of dynamic behaviours through the incorporation of kinetic information may help enhance the predictive power of the analyses and simplify the experimental effort put into reaching stationary stages.

## Supplementary Information

Below is the link to the electronic supplementary material.

Supplementary file1 (PDF 705 KB)

## Data Availability

The datasets generated during and analysed during the current study are available from the corresponding author on reasonable request.
