# Supplementary material for: Metabolic control analysis enabled the improvement of the L-cysteine production process with Escherichia coli
Source: Appl Microbiol Biotechnol. 2024 Jan 11;108(1):108. doi: 10.1007/s00253-023-12928-z (PMC10784400; doi:10.1007/s00253-023-12928-z)
Supplement: Supplementary file 1 — Supplementary file1 (PDF 705 KB) [file 253_2023_12928_MOESM1_ESM.pdf]

Supplementary Information

Metabolic Control Analysis Enabled the Improvement of the L-Cysteine Production Process with *Escherichia coli*

Daniel Alejandro Caballero Cerbon, Jeremias Widmann, Dirk Weuster-Botz

Technical University of Munich, School of Engineering and Design, Chair of Biochemical Engineering, Boltzmannstr. 15, 85748 Garching, Germany

(Corresponding author: dirk.weuster-botz@tum.de)

A

B

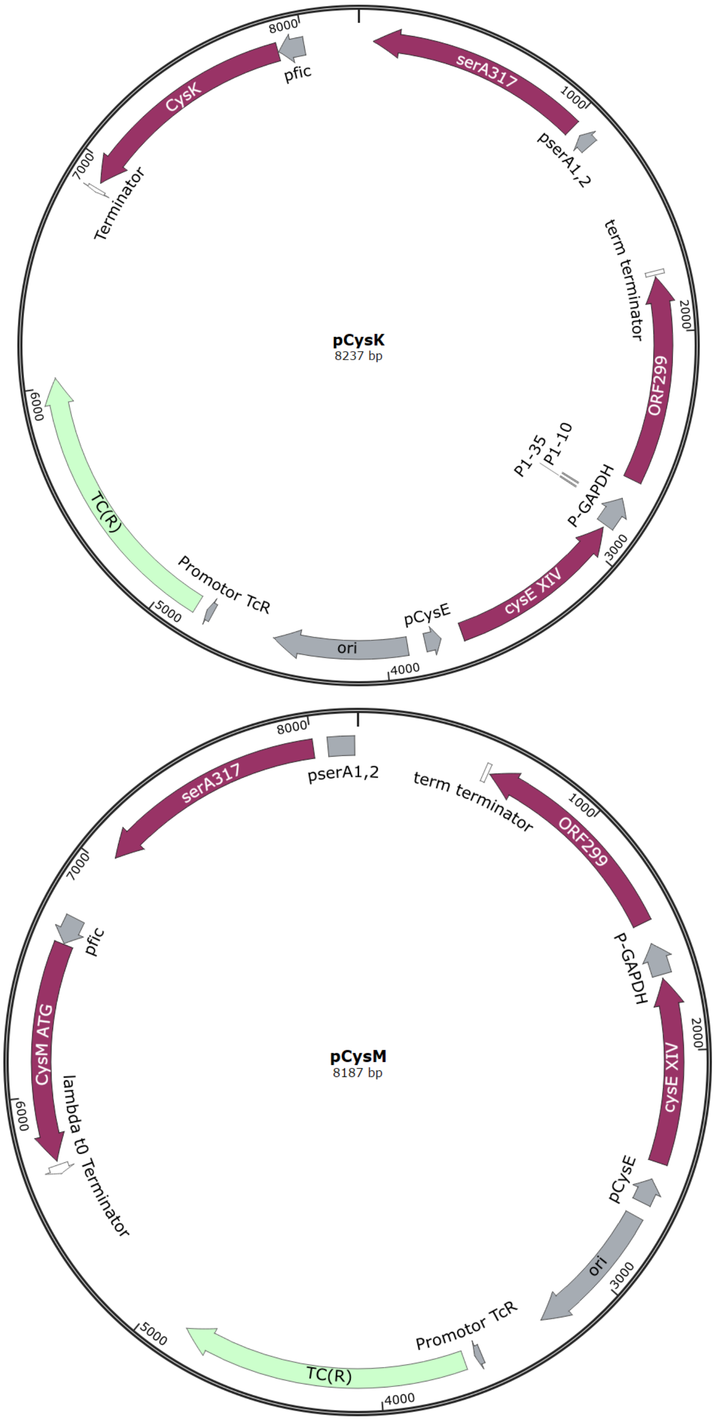

Figure S1: Plasmid maps of the novel plasmids pCysK (A) and pCysM (B)

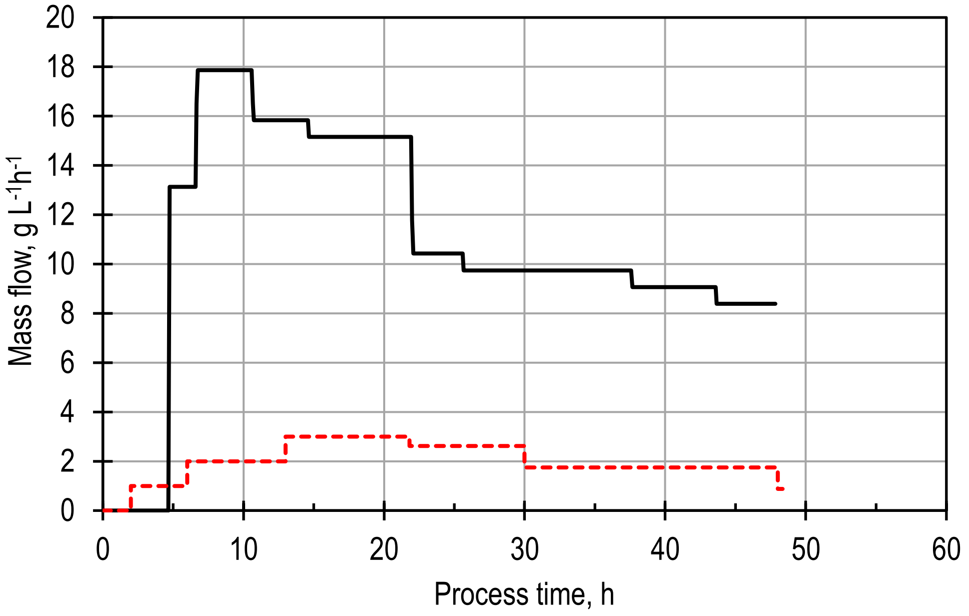

Figure S2: Glucose (continuous) and thiosulfate (dashed) feeding rate profiles for the L-cysteine production process on a 15 L scale. The rates refer to an initial reactor volume of 10 L.

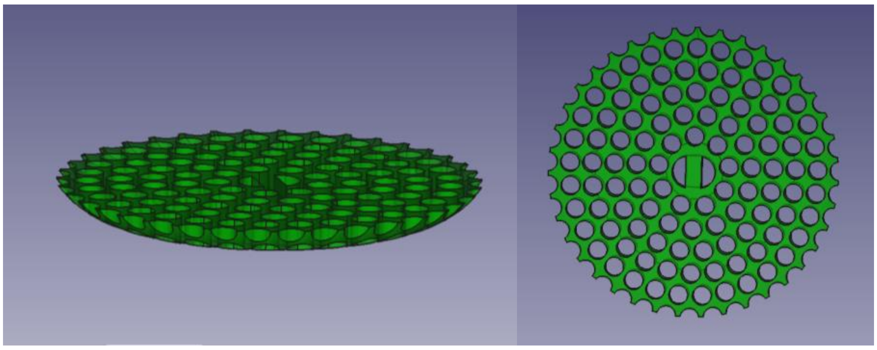

Figure S3: Side (left) and top (right) views of the centrifuge accessory designed for the efficient separation of the L-cystine and biomass pellets during the rapid media transition methodology. The piece is designed to fit at the bottom of 1 L centrifuge flasks (4239, Hettich, Tuttlingen, Germany). It has a total diameter of 12.5 cm and an individual hole diameter of 6 mm.

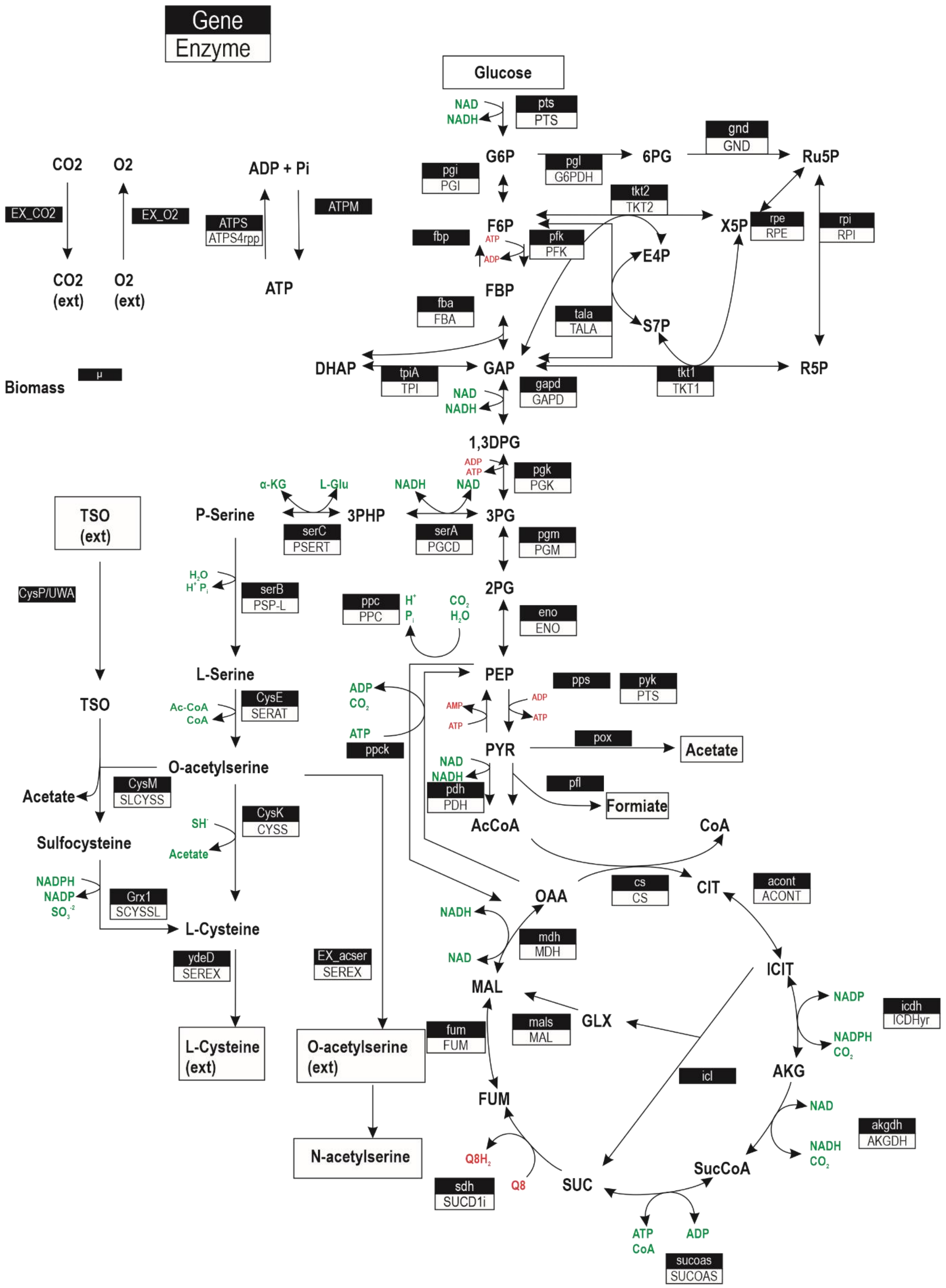

Figure S4: Reduced metabolic model used for the MCA. The gene identifiers are shown in black background whereas the enzyme identifiers are presented in white background. The metabolites presented in boxes with white background are extracellular.

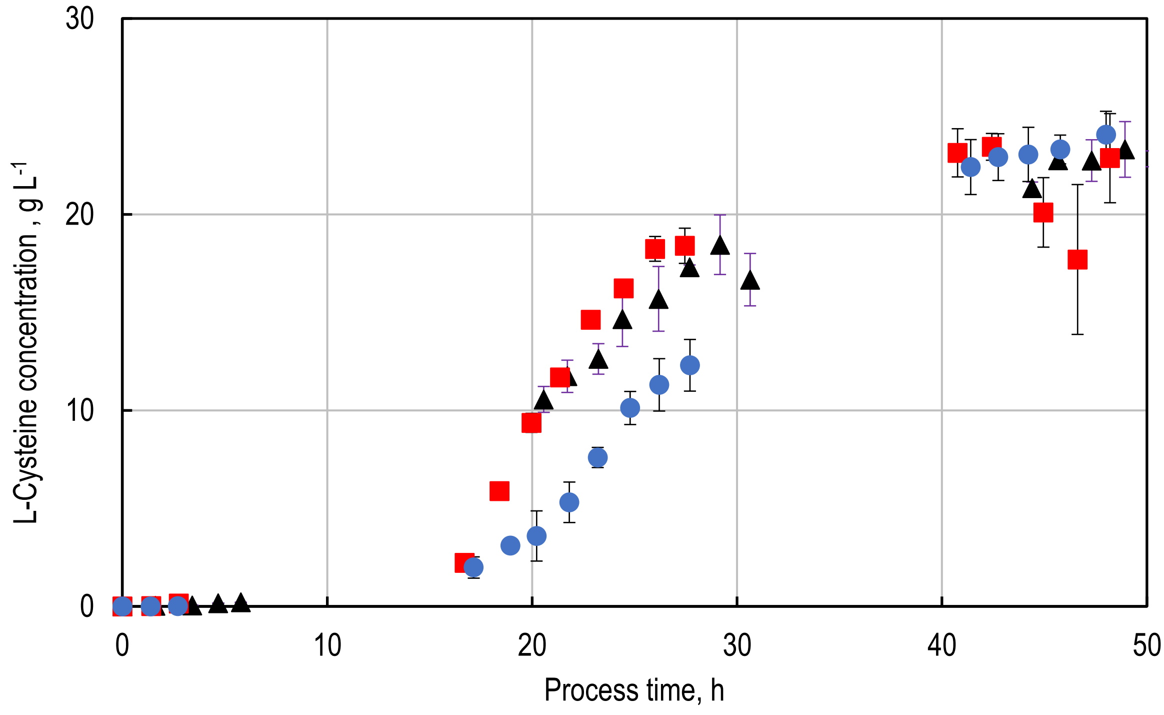

Figure S5: Biological replicates of the L-cysteine production process on a 15 L-scale with *E. coli* W3110 pCysK. In addition to the features of pCys, the plasmid pCysK contains the sequence for the overexpression of CYSS. The process in black is shown in figure 5 of this publication.

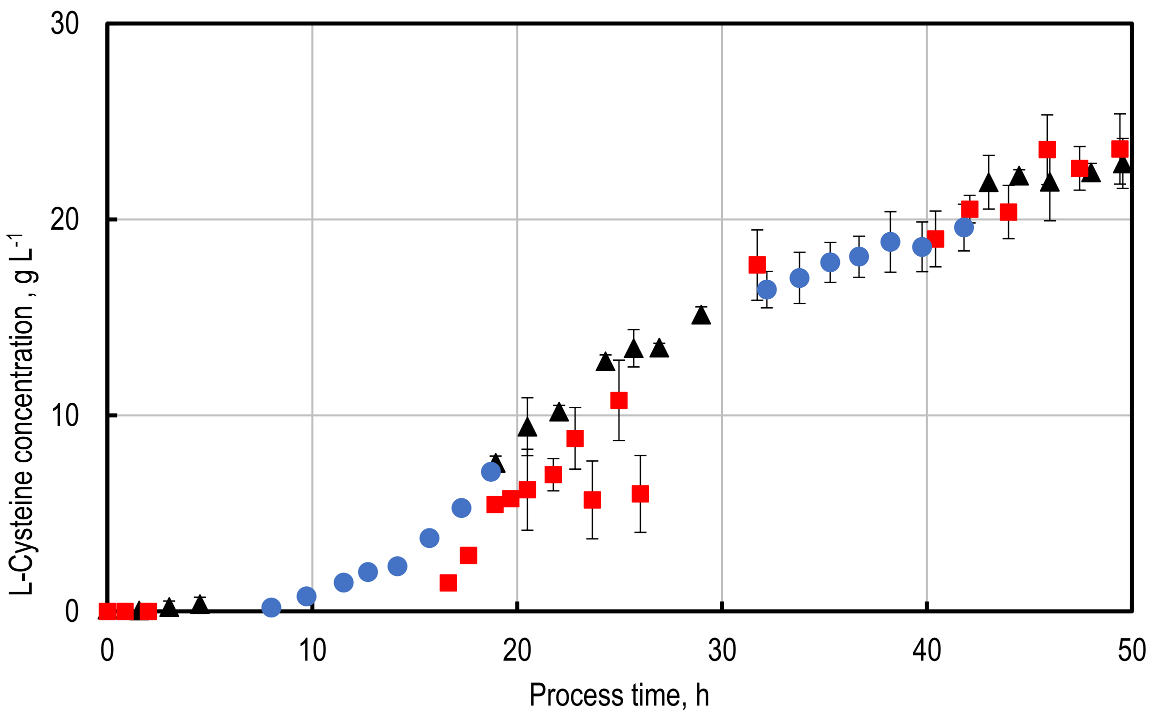

Figure S6: Biological replicates of the L-cysteine production process in 15 L-scale with *E. coli* W3110 pCysM. In addition to the features of pCys, the plasmid pCysK contains the sequence for the overexpression of CYSS. The process in blue was deliberately sampled in a shifted schedule when compared to the other processes in order to observe the behaviour of the L-cysteine concentration profile throughout the production process. The process in black is shown in figure 5 of this publication.

Table S1: Internal metabolite concentrations obtained from the LC-MS determination. The concentrations are given in mmol per litre cell volume. R.P.: Reference Process, Gluc: Parallel reactor supplied with glucose, Pyr: parallel reactor supplied with pyruvic acid, G+P: parallel reactor supplied with glucose and pyruvic acid, G+S: Parallel reactor supplied with glucose and succinate. U.b.:Upper boundary, L.b.: Lower boundary.

Metabolite	mmol/L	R.P.	Gluc1	Gluc2	Gluc3	Pyr1	Pyr2	Pyr3	G+P1	G+P2	G+P3	G+S1	G+S2	G+S3
**L-Glutamate**	L.b.	N.a.	126.0	119.2	112.06	74.62	57.36	109.0	103.3	91.5	140.0	89.64	110.2	154.1
	U.b.	N.a.	190.5	178.5	217.38	141.2	96.44	149.3	187.8	165.0	252.0	125.3	154.0	222.5
**Sulfocysteine**	L.b.	9.799	2.758	2.874	3.953	1.698	4.637	3.162	3.157	3.166	4.742	2.035	5.104	4.787
	U.b.	35.840	9.017	13.34	11.565	7.926	12.66	13.85	8.517	11.46	10.74	19.06	15.06	13.95
**G6P**	L.b.	0.284	0.261	0.474	0.459	0.210	0.338	0.293	0.279	0.445	0.795	0.618	0.444	0.262
	U.b.	0.644	0.358	0.594	0.709	0.269	0.438	0.394	0.436	0.568	1.198	0.870	0.566	0.347
**S7P**	L.b.	0.195	0.190	0.201	0.172	0.197	0.439	0.186	0.158	0.150	0.189	0.616	0.205	0.205
	U.b.	0.212	0.273	0.280	0.256	0.286	0.602	0.277	0.243	0.227	0.265	0.833	0.309	0.283
**F6P**	L.b.	0.697	0.493	0.726	0.403	0.566	1.276	0.551	0.496	0.474	0.499	1.862	0.601	0.675
	U.b.	1.047	1.190	1.296	1.011	0.899	1.943	0.967	0.831	0.889	0.906	2.821	0.958	1.040
**Ru5P**	L.b.	0.181	0.172	0.179	0.137	0.155	0.385	0.179	0.145	0.138	0.148	0.552	0.167	0.177
	U.b.	0.330	0.291	0.295	0.275	0.261	0.649	0.336	0.248	0.251	0.271	0.860	0.308	0.311
**P-ser**	L.b.	12.029	6.945	11.04	12.165	4.870	6.177	7.409	6.675	9.902	15.62	9.275	8.002	9.582
	U.b.	28.021	8.475	13.00	15.016	6.498	9.492	10.69	8.046	13.38	22.57	10.76	11.44	12.59
**NAD**	L.b.	0.891	0.779	0.811	0.708	0.566	0.779	0.761	0.628	0.751	0.798	1.094	0.973	0.983
	U.b.	1.059	1.137	1.310	1.149	0.878	1.047	0.976	1.051	1.112	1.360	1.303	1.230	1.166
**AMP**	L.b.	0.084	0.319	0.534	0.301	0.246	0.310	0.420	0.315	0.304	0.637	0.510	0.638	0.540
	U.b.	0.516	0.484	1.088	0.812	0.366	0.445	0.630	0.513	0.626	0.790	0.567	0.955	0.667
**Malate**	L.b.	0.020	0.289	0.198	0.218	0.265	0.616	0.543	0.159	0.272	0.777	1.095	0.571	0.163
	U.b.	0.657	0.601	0.445	0.442	0.620	0.860	1.044	0.518	0.560	1.807	1.400	0.945	0.540
**3PG**	L.b.	0.130	0.833	0.961	0.805	0.800	1.215	0.840	0.666	0.747	0.876	1.919	0.816	0.848
	U.b.	2.623	1.578	1.904	1.818	1.211	2.276	1.859	1.385	1.761	2.005	4.036	1.905	1.641
**6PG**	L.b.	2.736	2.491	2.473	2.165	2.421	5.792	2.597	2.130	2.051	2.327	7.963	2.641	2.747
	U.b.	3.185	3.277	3.249	2.825	3.169	7.583	3.431	2.908	2.824	3.214	10.05	3.367	3.518
**NADP**	L.b.	0.416	0.390	0.382	0.336	0.357	0.736	0.383	0.341	0.337	0.395	1.000	0.446	0.448
	U.b.	0.463	0.505	0.490	0.437	0.422	0.946	0.494	0.447	0.452	0.519	1.248	0.537	0.534
**PEP**	L.b.	0.281	0.311	0.344	0.285	0.291	0.624	0.332	0.276	0.233	0.296	0.907	0.335	0.308
	U.b.	0.471	0.464	0.473	0.454	0.429	0.964	0.468	0.447	0.446	0.515	1.279	0.561	0.438
**ADP**	L.b.	0.765	0.849	0.932	0.878	0.671	1.553	0.768	0.596	0.730	0.871	2.117	1.137	1.074
	U.b.	1.200	1.337	1.469	1.405	1.095	2.640	1.349	1.069	1.135	1.393	3.085	1.544	1.481
**FBP**	L.b.	0.152	0.369	0.388	0.343	0.376	0.778	0.407	0.329	0.328	0.404	1.195	0.426	0.412
	U.b.	0.313	0.492	0.505	0.447	0.514	1.057	0.548	0.456	0.449	0.516	1.571	0.543	0.535
**Cit/ICIT**	L.b.	11.373	17.18	17.19	6.468	4.456	10.19	16.86	6.284	12.08	14.28	23.96	9.123	16.06
	U.b.	32.276	35.74	44.54	44.227	41.04	43.11	37.53	39.51	42.27	60.64	60.16	52.13	49.11
**NADH**	L.b.	0.303	0.319	0.238	0.212	0.264	0.751	0.275	0.215	0.203	0.238	1.147	0.294	0.218
	U.b.	0.459	0.469	0.455	0.385	0.420	1.201	0.473	0.395	0.351	0.465	1.691	0.458	0.413
**FAD**	L.b.	0.158	0.242	0.249	0.209	0.247	0.532	0.262	0.213	0.208	0.237	0.771	0.275	0.272
	U.b.	0.207	0.316	0.325	0.273	0.318	0.699	0.343	0.286	0.287	0.319	0.983	0.346	0.343
**ATP**	L.b.	1.810	2.579	2.030	1.927	2.389	4.960	2.218	1.954	2.084	2.235	7.228	2.288	2.561
	U.b.	2.426	3.402	3.284	2.705	3.388	6.556	2.828	2.608	2.874	3.114	8.986	2.865	3.122
**NADPH**	L.b.	0.767	1.364	1.281	0.988	1.260	2.551	1.507	1.210	1.239	1.445	3.795	1.747	1.582
	U.b.	1.031	1.624	1.910	1.262	1.617	3.467	1.832	1.477	1.589	1.821	5.050	2.121	1.870
**AcCoA**	L.b.	1.958	2.131	2.556	2.222	2.111	4.592	2.208	1.881	2.283	2.761	5.645	2.109	2.077
	U.b.	2.458	2.661	3.015	2.769	2.579	5.825	2.893	2.292	2.752	3.433	7.194	2.559	2.498

Table S2: Metabolic fluxes obtained from the TFA. The rates are given in mmol per gram cell mas per hour. R.P.: Reference Process, Gluc: Parallel reactor supplied with glucose, Pyr: parallel reactor supplied with pyruvic acid, G+P: parallel reactor supplied with glucose and pyruvic acid, G+S: Parallel reactor supplied with glucose and succinate. U.b.:Upper boundary, L.b.: Lower boundary.

Reaction		R. P.	Gluc1	Gluc2	Gluc3	Pyr1	Pyr2	Pyr3	G+P 1	G+P 2	G+P 3	G+S 1	G+S 2	G+S 3
PTS	U.b.	0.961	0.328	0.653	1.058	0.000	0.000	0.000	0.366	0.726	1.173	0.228	0.452	0.476
	L.b.	0.890	0.326	0.570	0.924	0.000	0.000	0.000	0.301	0.596	0.964	0.203	0.427	0.424
PGI	U.b.	0.953	0.000	0.647	1.049	-0.002	-0.004	-0.006	0.363	0.717	1.158	-0.115	0.446	-0.457
	L.b.	0.005	0.000	0.000	0.000	-0.072	-0.139	-0.185	0.000	0.000	0.000	-0.433	0.000	-0.876
PFK	U.b.	0.920	0.212	0.615	1.000	0.000	0.000	0.000	0.345	0.671	1.063	0.002	0.410	0.008
	L.b.	0.475	0.211	0.189	0.478	0.000	0.000	0.000	0.149	0.146	0.194	0.000	0.090	0.000
FBA	U.b.	0.920	0.212	0.615	1.000	0.000	0.000	0.000	0.345	0.671	1.063	0.002	0.410	0.008
	L.b.	0.475	0.211	0.189	0.478	-0.102	-0.198	-0.263	0.149	0.146	0.194	0.000	0.090	0.000
TPI	U.b.	0.914	0.210	0.610	0.992	-0.014	-0.026	-0.035	0.342	0.664	1.050	0.000	0.405	0.000
	L.b.	0.468	0.210	0.184	0.347	-0.222	-0.428	-0.570	0.146	0.138	0.180	-0.002	0.084	-0.008
GAPD	U.b.	1.815	0.527	1.214	1.976	-0.031	-0.060	-0.080	0.681	1.320	2.020	0.216	0.781	0.436
	L.b.	1.190	0.525	0.706	1.197	-0.239	-0.461	-0.615	0.368	0.354	0.466	0.141	0.221	0.239
PGK	U.b.	1.815	0.527	1.214	1.976	-0.031	-0.060	-0.080	0.681	1.320	2.020	-0.141	-0.221	0.436
	L.b.	1.190	0.525	0.706	1.197	-0.239	-0.461	-0.615	0.368	0.354	0.466	-0.216	-0.781	0.239
PGM	U.b.	1.545	0.383	1.103	1.741	-0.129	-0.152	-0.490	0.332	0.901	1.543	0.247	0.250	0.243
	L.b.	0.920	0.380	0.594	0.961	-0.382	-0.639	-1.385	-0.030	-0.170	0.026	0.115	0.110	0.046
ENO	U.b.	1.545	0.383	1.103	1.741	-0.129	-0.152	-0.490	0.332	0.901	1.543	0.247	0.250	0.243
	L.b.	0.920	0.380	0.594	0.961	-0.382	-0.639	-1.385	-0.030	-0.170	0.026	0.115	0.110	0.046
PDH	U.b.	0.997	0.310	0.994	1.574	0.825	1.736	2.587	0.759	2.104	3.394	0.247	0.250	0.510
	L.b.	0.100	0.201	0.138	0.147	0.540	1.186	0.100	0.333	0.100	1.397	0.115	0.110	0.172
PPC	U.b.	0.327	0.093	0.248	0.445	0.000	0.000	0.000	0.450	0.000	0.000	0.237	0.206	0.000
	L.b.	0.185	0.049	0.000	0.000	0.000	0.000	0.000	0.123	0.000	0.000	0.172	0.067	0.000
CS	U.b.	1.068	0.111	0.812	1.236	0.760	1.554	2.139	0.271	1.452	2.255	0.072	0.169	0.264
	L.b.	0.054	0.037	0.049	0.076	0.426	0.960	1.224	0.090	0.264	0.477	0.000	0.000	0.041
ACONT	U.b.	1.068	0.111	0.812	1.236	0.760	1.554	2.139	0.271	1.452	2.255	0.072	0.169	0.264
	L.b.	0.054	0.037	0.049	0.076	0.426	0.960	1.224	0.090	0.264	0.477	0.000	0.000	0.041
ICDH	U.b.	1.055	0.030	0.812	1.236	0.727	1.491	2.055	0.271	1.322	1.904	0.072	0.169	0.264
	L.b.	0.054	0.029	0.049	0.076	0.357	0.808	1.129	0.074	0.071	0.126	0.000	0.000	0.041
AKGDH	U.b.	1.003	0.004	0.778	1.184	0.715	1.467	2.024	0.158	1.274	1.819	0.039	-0.111	0.236
	L.b.	0.017	0.004	0.016	0.024	0.195	0.464	0.688	0.009	0.023	0.041	-0.101	-0.615	0.013
SUCOAS	U.b.	0.985	0.001	0.763	1.160	0.709	1.456	2.009	0.149	1.251	1.778	0.101	0.615	0.222
	L.b.	0.005	0.000	0.000	0.000	0.189	0.453	0.673	0.000	0.000	0.000	-0.039	0.111	0.000
SUCDH	U.b.	1.022	0.000	0.779	1.184	0.747	1.530	2.108	0.054	1.404	2.048	0.272	0.765	0.709
	L.b.	0.017	0.000	0.016	0.024	0.378	0.847	1.182	0.000	0.194	0.270	0.000	0.125	0.423
FUM	U.b.	1.067	0.000	0.811	1.234	0.759	1.553	2.139	0.000	1.543	2.337	0.272	0.765	0.818
	L.b.	0.051	0.000	0.047	0.072	0.389	0.869	1.211	-0.046	0.239	0.349	0.000	0.125	0.449
MDH	U.b.	1.191	0.082	0.901	1.372	0.792	1.616	2.223	0.149	1.978	2.994	0.152	0.671	0.808
	L.b.	0.051	0.007	0.047	0.072	0.458	1.021	1.306	-0.046	0.391	0.700	0.012	0.034	0.368
G6PDH	U.b.	0.953	0.326	0.647	1.049	0.054	0.105	0.139	0.363	0.717	1.158	0.152	0.671	1.347
	L.b.	0.005	0.325	0.000	0.000	0.000	0.000	0.000	0.000	0.000	0.000	0.012	0.034	0.862
GND	U.b.	0.953	0.326	0.647	1.049	0.054	0.105	0.139	0.363	0.717	1.158	0.152	0.671	1.347
	L.b.	0.005	0.325	0.005	0.005	0.005	0.005	0.005	0.005	0.005	0.005	0.012	0.034	0.862
RPE	U.b.	0.607	0.212	0.408	0.663	0.028	0.053	0.071	0.229	0.444	0.712	0.144	0.637	0.879
	L.b.	-0.194	0.211	-0.127	-0.193	-0.081	-0.157	-0.209	-0.035	-0.263	-0.459	0.004	0.000	0.476
RPI	U.b.	0.441	0.114	0.324	0.506	0.099	0.191	0.254	0.209	0.566	0.952	0.000	0.026	0.468
	L.b.	0.024	0.113	0.028	0.043	0.004	0.008	0.010	0.016	0.040	0.072	-0.140	-0.621	0.347
TKT1	U.b.	0.345	0.108	0.241	0.385	0.045	0.086	0.115	0.150	0.346	0.571	0.430	1.069	0.445
	L.b.	0.005	0.107	0.000	0.000	-0.038	-0.074	-0.099	0.000	0.000	0.000	0.251	0.374	0.243
TKT2	U.b.	0.296	0.105	0.198	0.322	0.012	0.022	0.030	0.111	0.213	0.340	0.438	1.173	0.434
	L.b.	0.005	0.104	-0.141	-0.214	-0.066	-0.127	-0.170	-0.038	-0.263	-0.459	0.259	0.406	0.230

Table S2 (continuation): Metabolic fluxes obtained from the TFA. The rates are given in mmol per gram cell mas per hour. R.P.: Reference Process, Gluc: Parallel reactor supplied with glucose, Pyr: parallel reactor supplied with pyruvic acid, G+P: parallel reactor supplied with glucose and pyruvic acid, G+S: Parallel reactor supplied with glucose and succinate. U.b.:Upper boundary, L.b.: Lower boundary.

Reaction		R. P.	Gluc1	Gluc2	Gluc3	Pyr1	Pyr2	Pyr3	G+P 1	G+P 2	G+P 3	G+S 1	G+S 2	G+S 3
TALA	U.b.	0.343	0.107	0.240	0.383	0.044	0.086	0.114	0.149	0.344	0.568	0.438	0.949	0.444
	L.b.	0.000	0.107	-0.035	-0.053	-0.039	-0.075	-0.100	-0.008	-0.051	-0.090	0.233	0.122	0.240
ACS	U.b.	0.343	0.107	0.240	0.383	0.044	0.086	0.114	0.149	0.344	0.568	0.659	0.446	0.444
	L.b.	0.000	0.107	-0.035	-0.053	-0.039	-0.075	-0.100	-0.008	-0.051	-0.090	0.341	0.000	0.240
PGCD	U.b.	0.824	0.146	0.537	0.881	0.334	0.546	1.263	0.627	1.259	0.621	0.659	0.446	0.246
	L.b.	0.270	0.145	0.112	0.236	0.098	0.092	0.410	0.349	0.335	0.355	0.341	0.000	0.193
PSERT	U.b.	0.824	0.146	0.537	0.881	0.334	0.546	1.263	0.627	1.259	0.621	0.434	0.274	0.246
	L.b.	0.270	0.145	0.112	0.236	0.098	0.092	0.410	0.349	0.335	0.355	0.117	-0.162	0.193
PSP	U.b.	0.824	0.146	0.537	0.881	0.334	0.546	1.263	0.627	1.259	0.621	-0.182	-0.028	0.246
	L.b.	0.270	0.145	0.112	0.236	0.098	0.092	0.410	0.349	0.335	0.355	-0.225	-0.340	0.193
SERAT	U.b.	0.374	0.136	0.389	0.578	0.253	0.254	0.774	0.503	0.595	0.516	0.218	0.203	0.212
	L.b.	0.219	0.135	0.069	0.172	0.083	0.063	0.371	0.332	0.275	0.251	0.060	0.000	0.159
CYSS	U.b.	0.177	0.040	0.151	0.195	0.172	0.011	0.218	0.146	0.129	0.178	0.215	0.131	0.099
	L.b.	0.005	0.005	0.005	0.005	0.005	0.005	0.005	0.005	0.005	0.005	0.057	-0.180	0.005
SLCYSS	U.b.	0.177	0.040	0.150	0.194	0.172	0.011	0.218	0.110	0.129	0.178	0.218	0.202	0.099
	L.b.	0.005	0.005	0.005	0.005	0.005	0.005	0.005	0.005	0.005	0.005	0.060	-0.036	0.005
SCYSSL	U.b.	0.177	0.040	0.150	0.194	0.172	0.011	0.218	0.110	0.129	0.178	0.164	0.311	0.099
	L.b.	0.005	0.005	0.005	0.005	0.005	0.005	0.005	0.005	0.005	0.005	0.000	0.000	0.005
SEREX	U.b.	0.199	0.123	0.243	0.390	0.133	0.247	0.597	0.316	0.165	0.328	0.312	0.143	0.208
	L.b.	0.128	0.096	0.005	0.059	0.005	0.058	0.280	0.270	0.005	0.147	0.165	-0.001	0.112
NADH5	U.b.	4.053	0.321	4.200	5.598	1.733	3.174	4.651	1.711	6.079	9.533	1.133	0.000	1.980
	L.b.	0.005	0.005	0.005	0.005	0.005	0.005	0.005	0.005	0.005	0.005	0.000	0.000	0.005
ATPS	U.b.	6.174	0.649	7.856	11.42	3.583	6.607	9.409	3.039	12.01	18.48	2.442	7.111	4.290
	L.b.	1.763	0.385	0.413	1.671	0.850	1.060	2.028	1.291	1.474	2.754	0.736	1.369	1.968
